# Vaccination with a Paramyosin-Based Multi-Epitope Vaccine Elicits Significant Protective Immunity against *Trichinella spiralis* Infection in Mice

**DOI:** 10.3389/fmicb.2017.01475

**Published:** 2017-08-03

**Authors:** Yuan Gu, Ximeng Sun, Bo Li, Jingjing Huang, Bin Zhan, Xinping Zhu

**Affiliations:** ^1^Department of Medical Microbiology and Parasitology, School of Basic Medical Sciences, Capital Medical University Beijing, China; ^2^Section of Tropical Medicine, Department of Pediatrics, Baylor College of Medicine, Houston TX, United States

**Keywords:** *Trichinella spiralis*, Trichinellosis, multi-epitope, vaccine, paramyosin, protective immunity

## Abstract

Trichinellosis is a worldwide zoonosis and remains a serious public health problem. Interrupting parasite transmission via vaccination of livestocks with a potent vaccine is a practical approach to prevent human Trichinellosis. Our previous studies have identified that paramyosin of *Trichinella spiralis* (*Ts*-Pmy) is a good vaccine candidate against Trichinellosis. In this study, a novel multi-epitope vaccine (MEP) was constructed by using four CD4^+^ T cell epitopes (P2, P3, P4, and P5) and one B cell epitope (YX1) from *Ts*-Pmy and expressed as a soluble recombinant protein (rMEP) in *Escherichia coli*. Mice immunized with rMEP vaccine produced significant higher muscle larval reduction (55.4%) than that induced by immunization of parental r*Ts*-Pmy (34.4%) against *T. spiralis* infection. The better protection is associated with rMEP induced high levels of anti-rMEP specific IgG and subclass IgG1/IgG2a, elevated T cell proliferation of splenocytes and secretion of IFN-γ, IL-4 and IL-5. The cellular response to individual T cell epitope also showed that splenocytes from mice immunized with rMEP strongly response to the stimulation of synthetic epitope peptide P2, P3, and P4, but not to P5, suggesting that most of T cell epitopes are exposed and processed well during immunization that may contribute to the high protection induced by the immunization of rMEP. This study implies that epitope vaccine is a promising approach for the development of vaccines against Trichinellosis.

## Introduction

Trichinellosis is a worldwide food-borne zoonosis. The parasitic nematode *Trichinella spiralis*, one of the most common etiologic agents of Trichinellosis, infects both wild and domestic animals. Human infection is mainly acquired by ingesting raw or undercooked meat infected with *Trichinella* larvae and contaminated pork is the major infective source in China (Cui and Wang, 2011; Ortega-Pierres et al., 2015). Trichinellosis clinical manifestations range from asymptomatic infection to fatal disease (Wilson N.O. et al., 2015). This zoonosis is not only a serious public health problem to human but also causes a big economic loss for the meat processing industry as China is now the largest producer and consumer of pork in the world (Dobrescu et al., 2014). Therefore, to develop a vaccine preventing swine infection would make a feasible contribution for the control of Trichinellosis.

During the past decades, many vaccine candidates against Trichinellosis have been investigated and reported, including those based on crude larval extracts (Deville et al., 2005), excretory-secretory (ES) products (Dea-Ayuela et al., 2006), DNA (Wang et al., 2016), recombinant proteins (Bi et al., 2015), peptides (Castillo et al., 2013) or combined DNA with protein (Gu et al., 2014), all of them induced different extents of partial protective immunity in animal models. However, due to the complexity of the life cycle, diversity of stage-specific antigens and immune-evasion strategies of *T. spiralis* (Zhang et al., 2011; Sun et al., 2015), researchers face the challenges in developing effective vaccines against Trichinellosis. Subunit vaccine based on multiple protective epitopes of several vaccine antigens possibly enables investigators to overcome these problems and provides a novel approach to develop vaccines against infectious diseases (Cao et al., 2015; Meza et al., 2017).

Paramyosin (Pmy) is a thick myofibrillar protein found only in invertebrates (Gobert and McManus, 2005). Pmy is not only a structural component of myofilament but also a promising vaccine candidate in some helminths such as *Schistosoma mansoni* (Ramirez et al., 1996), *Brugia malayi* (Li et al., 1993) and *Taenia solium* (Vazquez-Talavera et al., 2001). Our previous studies have identified that paramyosin of *T. spiralis* (*Ts-*Pmy) is a good vaccine candidate against *Trichinella* infection in term of its potential to induce protective immunity against the challenge of *T. spiralis* larvae (Yang et al., 2008; Yang J. et al., 2010). However, difficulty in expressing *Ts*-Pmy as a soluble rMEP due to its large molecular weight (102 kDa) prevents it from being scaled up for vaccine purpose. In addition, the identified complement-binding activities of recombinant *Ts*-Pmy protein raise a concern of possible interference of human normal complement function when *Ts*-Pmy is administered as a vaccine (Zhang et al., 2011; Sun et al., 2015). Compared to whole-protein antigens, an epitope-based vaccine may overcome these above-mentioned shortcomings and have many advantages such as increased safety and immunogenicity of vaccine for increased protective potency (Tang et al., 2015). The epitope-based vaccines are on the road to the market, especially in the treatment of cancer (Higashihara et al., 2014; Correale et al., 2016).

In our previous study, in an effort to understand immunoprotective mechanism of *Ts*-Pmy, a protective B cell epitope named YX1, including 20 amino acids within *Ts*-Pmy 88-107, has been identified to be recognized by a monoclonal antibody (mAb) 7E2 which protects passively transferred mice from being infected with *T. spiralis* (Wei et al., 2011). In addition, four potent CD4^+^ T cell epitopes of *Ts-*Pmy that could strongly induce both Th1 and Th2 immune responses were also identified (Gu et al., 2016). These results make it possible to design an effective epitope-based subunit vaccine against Trichinellosis. In this study a multi-epitope protein (MEP) vaccine was constructed based on previously identified B and T cell epitopes and its immunogenicity and vaccine efficacy was evaluated in a mouse model.

## Materials and Methods

### Ethics Statement

This study was performed in accordance with the National Institutes of Health Guidelines for the Care and Use of Experimental Animals. All animal experimental procedures were reviewed and approved by the Medicine Animal Care and Use Committee of Capital Medical University (approval number: AEEI-2015-149).

### Mice and Parasites

Female, 6–7 week old BALB/c mice were provided by the Laboratory Animal Services Center of Capital Medical University (Beijing, China) and raised under specific-pathogen-free standard conditions.

The *T. spiralis* (ISS 533) strain used in this study was obtained from a swine source in the Heilongjiang province, China. Serial passages were maintained in female ICR mice. Each mouse was orally challenged with 400 *T. spiralis* infective muscle larvae (ML). Six weeks after challenge, the ML were collected from the muscle tissue of infected mice and digested with 1% pepsin and 1% HCl (Gamble et al., 2000; Gu et al., 2013).

### Construction of Multiple-Epitope Vaccine (MEP)

**FIGURE 1:**
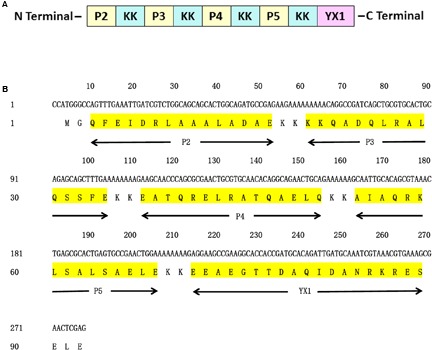
Design of MEP gene. **(A)** Schematic representation of T cell epitopes, B cell epitope of *Ts*-Pmy and spacers (KK). **(B)** Nucleotide and amino acid sequences of MEP. The position of each T cell epitope, B cell epitope and the spacer (KK) is indicated below the amino acid sequence.

In order to enhance Th1 and Th2 responses of vaccine candidate *Ts*-Pmy, a multiple-epitope vaccine (MEP) was constructed based on previously identified four T cell epitopes (P2, P3, P4, and P5) (Gu et al., 2016) and one B cell epitope YX1 (Wei et al., 2011). The MEP vaccine was designed by arranging the five epitopes in the order of following sequence P2 – P3 – P4 – P5 – YX1. To minimize interference between adjacent epitopes, each was separated from its neighboring epitope by a bi-lysine (KK) spacer (Yano et al., 2003). The construction of MEP array and sequence is illustrated in **Figure 1**. The DNA encoding MEP (278 bp) was chemically synthesized by Invitrogen Biotechnology (Shanghai, China) and cloned into the bacterial expression plasmid vector pET28a (Novagen, United States) using *Nco* **I** and *Xho* **I** restriction sites with a reading frame of six histidine-tag expressed at C-terminus.

### Expression and Purification of rMEP

The recombinant pET28a-MEP was transformed into *Escherichia coli* BL21 and recombinant MEP protein (rMEP) was expressed under induction of IPTG at a final concentration of 1 mM at 37°C for 4 h. The rMEP with six His-tag was expressed as a soluble protein and purified by Ni-affinity chromatography (Novagen, Germany). The purified rMEP was transferred on NC membrane (Millipore, United States) and probed with an anti-His tag mAb (1:5000) or 7E2 (a mAb against the B cell epitope YX1 of *Ts*-Pmy, 1:5000) (Wei et al., 2011). IRDye 680LT -conjugated goat anti-mouse IgG was added as the secondary antibody and the binding was visualized by an Odyssey infrared imaging system (Li-Cor, United States).

### Immunization Regimen

Three groups of female BALB/c mice (12 animals per group) were included in this study. One group received three doses of 25 μg of purified rMEP protein emulsified with the water-in-oil adjuvant ISA 50V2 (SEPPIC, France) in a total volume of 100 μl at an interval of 2 weeks. Another two groups of mice immunized with recombinant *Ts*-Pmy protein (r*Ts*-Pmy) or PBS at the same regimen were served as controls. One week after the final boost, six mice from each group were sacrificed for collecting sera and spleens to evaluate the induced humoral and cellular immune responses.

### Detection of Antibody Responses

The levels of the antigen-specific total IgG, IgG1 and IgG2a antibodies in sera of immunized mice were determined by ELISA. Briefly, 96-well microtiter plates (Costar) were coated with rMEP or r*Ts*-Pmy (1 μg/mL) and blocked with PBS containing 1% bovine serum albumin (BSA). To detect the total IgG, serum samples at different dilutions were added to each well and then incubated with an HRP-conjugated goat anti-mouse IgG. To detect IgG1 and IgG2a isotype, the mouse sera with 1: 200 dilution were added to each well and then incubated with Biotin Rat Anti-Mouse IgG1 or IgG2a (BD Pharmingen, United States) followed by Streptavidin-HRP (BD Biosciences, United States). The color was developed with tetramethylbenzidine (TMB) substrate (BD Biosciences, United States) and read at 450 nm.

### T Cell Proliferation

T cell proliferation assays were performed using the CellTiter 96^®^AQ_ueous_One Solution Cell Proliferation Assay (Promega, United States). Briefly, splenocytes were isolated from spleens of immunized mice (Dakewe, China) and 5 × 10^5^ splenocytes were stimulated with individual epitope peptide (5 μg/mL) or rMEP (10 μg/mL) for 72 h *in vitro*. Culture media without any antigen was served as negative control. Finally, 40 μL of the One Solution Reagent was added to each well and incubated for additional 1–4 h at 37°C. The stimulation index (SI) was calculated as the ratio of the mean OD value of antigen-stimulated wells to the mean OD value of medium-stimulated wells.

### Cytokine Analysis

IFN-γ, IL-2, IL-4, and IL-5 secreted by splenocytes isolated from the immunized mice were detected by an enzyme-linked immunospot assay (ELISPOT, BD Biosciences, United States). Briefly, the mice were sacrificed 7 days after the final immunization and splenocytes were separated aseptically using mouse lymphocyte separation medium (Dakewe Biotech, China). The 96-well plates for ELISPOT (Millipore, United States) were coated with the capture antibody (anti-mouse IFN-γ, IL-2, IL-4, and IL-5; BD Biosciences, United States) diluted in PBS (1:200) at 4°C overnight and then blocked with RPMI 1640 medium (Gibco, United States) containing 10% FBS for 2 h at room temperature. For IL-2, IL-4, and IL-5 detection, a total of 1 × 10^6^ lymphocytes were added to each well and 5 × 10^5^ lymphocytes were added for IFN-γ detection. The lymphocyte cells were stimulated with rMEP and individual epitope peptides at a final concentration of 5 μg/mL for 48 h. Cells stimulated with Concanavalin A (ConA, Sigma, United States; 5 μg/mL) were used as a positive control. A total of 100 μL biotinylated detection antibody (anti-IFN-γ, IL-2, IL-4, and IL-5 antibody; BD Biosciences, United States) diluted in PBS containing 10% FBS (1: 200) was added and incubated for 2 h. Then the wells were incubated with 100 μL of Streptavidin-HRP for 1 h (BD Biosciences, United States) and finally the color was developed with 100 μL of substrate solution (BD ELISPOT AEC substrate set; BD Biosciences, United States) for 1–5 min. The spot-forming units (SFU), which represent cells that have secreted cytokine in response to antigen stimulation during the assay, were counted with a CTL ELISPOT reader and analyzed using the ImmunoSpot image analyzer software v4.0.

### Evaluation of Larval Burden

One week after the third immunization, the left six mice of each group were infected orally with 400 *T. spiralis* infective ML. Six weeks after the challenge, the larvae from the muscle of each infected mice were collected and counted (Gu et al., 2013). Three independent experiments were carried out and the data were shown as one representative experiment. The reduction rate in ML was calculated based on the recovered larvae per gram (LPG) muscle from the mice immunized with rMEP or r*Ts*-Pmy versus those from the PBS control group.

### Statistical Analysis

Statistical analyses were performed with One-way ANOVA using SPSS for Windows, version 17.0. All data are expressed as the mean value ± standard deviations (SD). Differences were considered significant at *p* < 0.05.

## Results

### Construction, Expression and Characterization of rMEP

**FIGURE 2:**
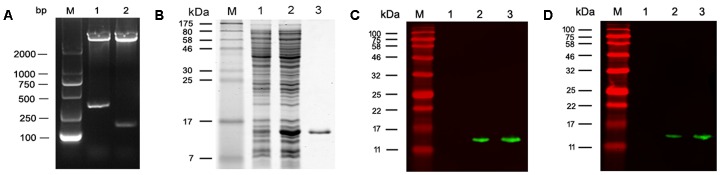
Expression and characterization of rMEP. **(A)** Restriction endonuclease digestion of recombinant plasmid pET28a-MEP. Recombinant plasmid pET28a-MEP was digested with *Nco* **I** and *Xho* **I** to release the insert. M: DL2000 DNA marker; lane 1: pET28a-MEP plasmid digested with *Nco* **I** and *Xho* **I**; lane 2: empty pET28a plasmid digested with *Nco* **I** and *Xho* **I**. **(B)** rMEP was highly expressed in *E. coli* BL21 lysates after IPTG induction analyzed by SDS - PAGE (10 μg, Lane 2), no expression of rMEP was shown in the uninduced *E. coli* BL21 lysate (10 μg, Lane 1); rMEP purified by Ni-affinity chromatography was loaded in Lane 3 (1 μg). **(C,D)** The expressed rMEP was detected by Western blot in induced lysate (1 μg, Lane 2) or purified rMEP (50 ng, Lane 3), but not detected in uninduced *E. coli* lysate (1 μg, Lane 1) by an anti-His tag monoclonal antibody **(C)** or by anti-B cell epitope YX1 of *Ts*-Pmy mAb 7E2 **(D)**.

The coding sequence of MEP was cloned into pET28a (+) and the recombinant plasmid pET28a-MEP was digested with *Nco* **I**/*Xho* **I** to release an insert of about 278 bp which corresponds with the size of MEP coding DNA (**Figure 2A**). rMEP was expressed in *E. coli* BL21 (DE3) as a soluble protein with approximately 11 kDa upon induction with IPTG. His-tagged rMEP was purified by Ni-NTA column chromatography (**Figure 2B**). Western blotting confirmed that the purified rMEP could be recognized by an anti-His antibody (**Figure 2C**) and anti-YX1 mAb 7E2 as well (**Figure 2D**).

### Serological Immune Response to rMEP Immunization

**FIGURE 3:**
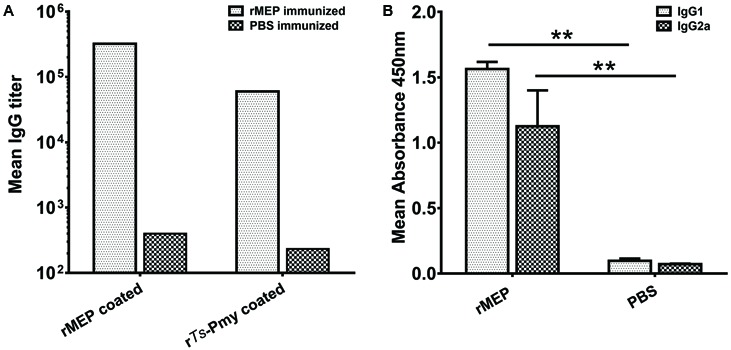
Serological antibody responses to immunization of rMEP measured by ELISA. **(A)** Specific IgG titers against rMEP or r*Ts*-Pmy in the sera of rMEP or PBS immunized mice were detected. The total IgG is shown as the geometric mean titer of six mice within the group (one representative experiment out of three). **(B)** The subtype IgG1 and IgG2a responses (OD at 1:200 dilutions) in the sera of rMEP or PBS immunized mice were detected. The values are shown as the mean absorbance of six mice ± SD (one representative experiment out of three). ^∗∗^*p* < 0.01.

The mice were immunized with rMEP for three times and the sera were collected 7 days after the final immunization. The antibody titers against rMEP or parental r*Ts*-Pmy were detected by ELISA. Anti-rMEP IgG levels in mice immunized with rMEP were greatly elevated and the mean IgG titer of six mice reached 1:320,000 after the third immunization. The rMEP immunized mouse sera also recognized r*Ts*-Pmy, the parental protein from which the MEP is designed, and the mean IgG titer of six mice against r*Ts*-Pmy reached 1:60,000 after the third immunization (**Figure 3A**). The anti-rMEP antibody titer remained 1:300,000 in mice 3 months post the final immunization (data not shown). The IgG antibody subclass determination verified that the predominant IgG subclass induced by rMEP was IgG1, but the IgG2a antibody response was also elevated (**Figure 3B**).

### T cell Proliferative Response to rMEP Immunization

**FIGURE 4:**
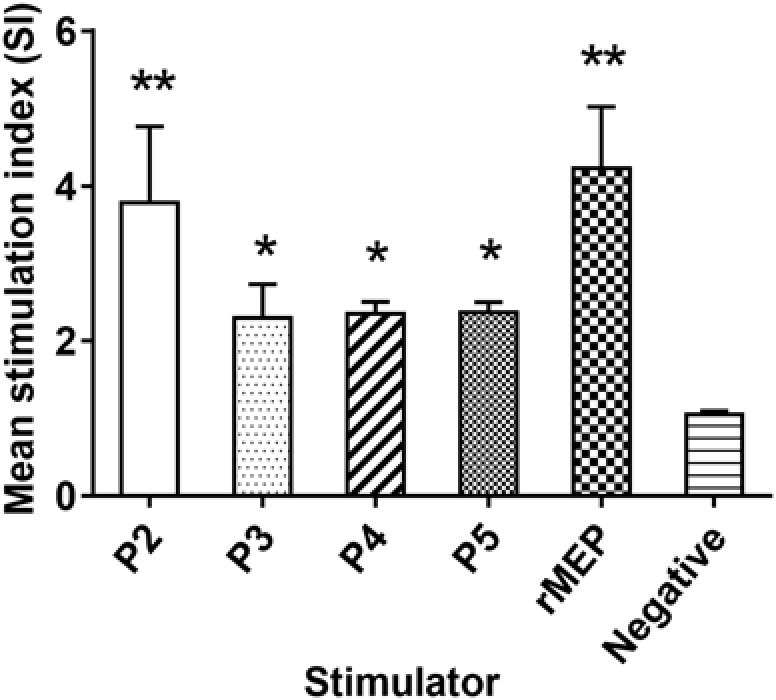
Proliferative responses of splenocytes isolated from the rMEP-immunized mice upon stimulation of individual T cell epitope peptide and rMEP *in vitro*. All four T cell epitope peptides and rMEP stimulated significantly higher T cell proliferations of splenocytes compared to the negative control (media only). The results are presented as the mean ± SD for six mice per group (one representative experiment out of three).*^∗∗^p* < 0.01, *^∗^p* < 0.05.

The lymphocytes were isolated from the spleen 1 week after the third immunization of rMEP. T cell proliferation results showed that rMEP, as well as four T cell epitope peptides, P2, P3, P4, and P5, all stimulated significantly T cell proliferative response than the negative control, with P2 producing the highest SI among the epitope peptides at the similar level as rMEP induced (^∗∗^*p* < 0.01 or ^∗^*p* < 0.05), indicating these four T cell epitopes of rMEP presented effectively *in vivo* immunization and stimulated cellular immune response (**Figure 4**).

### Splenocyte Cytokine Profiles

**FIGURE 5:**
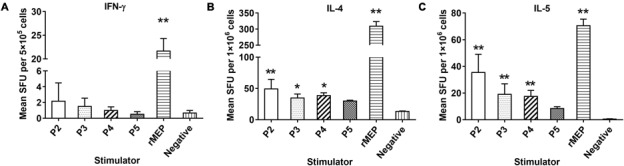
Cytokines secreted by splenocytes from rMEP-immunized mice upon stimulation of individual T cell epitope peptide and rMEP *in vitro*. Splenocytes secreting IFN-γ **(A)**, IL-4 **(B)** and IL-5 **(C)** were detected by ELISPOT 1 week after the final immunization. The results are shown as the mean ± SD.*^∗∗^p* < 0.01, *^∗^p* < 0.05.

The cytokines IFN-γ, IL-2, IL-4, and IL-5 secreted by immunized mouse splenocytes upon stimulation of rMEP or individual epitope peptides *in vitro*, were detected by ELISPOT. Th1 cytokines (IFN-γ) and Th2 cytokines (IL-4, IL-5) were significantly elevated in mice immunized with rMEP when stimulated with rMEP as compared with the PBS control group. For individual epitope peptide stimulation, only P2, P3, and P4 stimulated significantly higher Th2 cytokines IL-4 and IL-5 secretion compared to PBS control mice, but not as significantly high as rMEP stimulation. The secretion of IFN-γ was hardly tested in the splenocyte cells (less than 10 spots) when stimulated with P2, P3 and P4. P5 did not stimulate any of these four cytokines in rMEP immunized mice compared to PBS control mice. Meanwhile, neither rMEP nor each of four epitope peptides stimulated IL-2 secretion of splenocytes from rMEP immunized mice or PBS control mice. In the ConA stimulated positive control wells, the spots were all high up to 400/5 × 10^5^cells (data not shown). These results showed that rMEP vaccination induced a mixed Th1 and Th2 cytokine responses (IFN-γ, IL-4 and IL-5) in mice at the level much higher than the stimulation of each individual epitope peptide (**Figure 5**).

### Protective Immunity Elicited by rMEP Immunization

**FIGURE 6:**
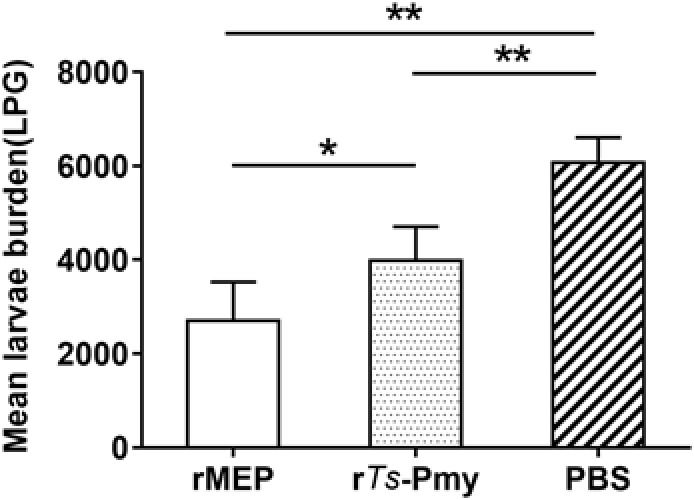
Protection elicited by immunization with rMEP. The larvae per gram muscle (LPG) were counted in the muscles of mice 42 days after a challenge with 400 *T. spiralis* muscle larvae. The results are shown as the mean of six mice in each group ± SD (one representative experiment out of three), ^∗∗^*p* < 0.01, ^∗^*p* < 0.05.

Mice immunized with rMEP produced 55.4% muscle larval reduction against *T. spiralis* infection compared to mice given with PBS as control (^∗∗^*p* < 0.01), which is significantly higher than the protection conferred by the parental r*Ts*-Pmy (34.4% larval reduction, **Figure 6**).

## Discussion

Immune-dominant epitopes of an antigen are very important in priming of the immune system efficiently as the immune response is always mounted against these regions. Therefore, immune-dominant epitopes, or collections of these epitopes, are considered as potential vaccine candidates (Rosa et al., 2010). Recombinant DNA technology has opens up a wide range of possibilities for designing a rational vaccine of chimeric proteins with several effective epitopes. Multi-epitope vaccines have the following advantages: several immune-protective epitopes can be included in a single immunogen, epitopes or regions without protective effect can be removed, and epitopes with adjuvant effects such as promiscuous T cell epitopes can be included to enhance protective efficacy (Fonseca et al., 2016).

Compared to vaccinating with the whole antigen, epitope-driven vaccine overcomes some potential safety concerns and appears to be capable of inducing more potent immune responses (Tang et al., 2015). To design a vaccine capable of inducing convenient humoral or cellular immune responses, it is essential to include the epitopes that could elicit the desired immune response (Soria-Guerra et al., 2015). The CD4^+^ T helper (Th) cells are activated when the peptide antigens are presented by MHC II molecules. Once activated, Th cells will divide rapidly and regulate the active immune response by secreting cytokines (Liu et al., 2011). As part of the adaptive immune response, CD4^+^ T cells play an important role in providing protective functions, including cytokine-mediated and contact-mediated signals to B cells, CD8^+^ T cells, and innate-immune cells, as well as direct attack on pathogens (Mettu et al., 2016). As CD4^+^ T cells are crucial in determining the functional status of both innate and adaptive immune responses, it is essential to comprise appropriate CD4^+^ T cell epitopes to improve the vaccine efficacy (Rosa et al., 2010). Many studies have shown that protective efficacy can be significantly improved by including an array of promiscuous T cell epitopes in vaccine constructs (Wen et al., 2014; Wu et al., 2016). Meanwhile, protective B-cell epitopes are also essential for developing epitope-based vaccines (Zhao et al., 2015; Sharma and Dixit, 2016). The multi-epitope vaccine is an attractive option and some epitope-based vaccines have been carried out in clinical trial against virus infection and carcinoma up to now (Lennerz et al., 2014; Nakamura et al., 2016).

Our previously identified B cell and T cell epitopes on a leading vaccine candidate *Ts*-Pmy laid a solid foundation for designing an effective epitope-based multivalent subunit vaccine against Trichinellosis. A novel multi-epitope vaccine called MEP was constructed in this study, which includes four T cell epitopes (P2, P3, P4, and P5) and one B cell epitope (YX1) of *Ts*-Pmy. The rationale for the design of MEP centers around two critical considerations. Firstly, a combination of T- and B-cell epitopes may enhance the induction of both cell-mediated and humoral immune responses. It has been proved that a mixed humoral and cellular immune response contributed to the protective immunity against *T. spiralis* infection (Bi et al., 2015; Liu et al., 2015). Secondly, the strategy involves positioning T cell epitopes on the N-terminal side and B-cell epitopes on the C-terminal side separated each epitope by a di-lysine linker. Some studies showed that the chimera with T cell epitope located in N-terminus and B cell epitope located in C-terminus induced higher affinity and specificity of antibody than did the reverse orientation (Partidos et al., 1992). The bi-lysine linker (KK) was inserted between different epitopes to preserve their independent immunological activities. KK is the target sequence of cathepsin B, which is one of the important proteases for antigen processing in the context of MHC-II antigen presentation (Sarobe et al., 1993). Splenocytes from mice immunized with rMEP showed increased T cell proliferations and cytokines secretion upon stimulation of individual T cell epitopes, P2, P3, and P4, but not by P5, indicating that most of the arranged epitopes were presented and processed correctly in immune response *in vivo*. B cell epitope constructed at the C-terminus of MEP vaccine could be recognized by the mAb 7E2 in Western Blot. It confirms that B cell epitope maintains its antigenicity in the MEP vaccine.

Mice immunized with *E. coli*-expressed soluble recombinant MEP protein elicited 55.4% muscle larval reduction against *T. spiralis* infection compared to mice administrated with PBS only, which is significant higher than 34.4% larval reduction induced by the parental r*Ts*-Pmy and reaches the highest protection so far among all DNA or recombinant protein vaccine candidates developed in our lab. In our previous study, mice vaccinated with r*Ts*-Pmy protein formulated with different adjuvants demonstrated a 33.7–36.7% ML burden reduction respectively against *T. spiralis* larvae challenge (Yang et al., 2008; Yang J. et al., 2010). Salmonella-delivered *Ts*-Pmy DNA vaccine also produced a significant 46.6% reduction in ML burden after challenge (Wang et al., 2016). Other vaccine candidates, for example, *Ts*-ES-1 and *Ts*87, were also investigated in our group. Recombinant *Ts*-ES-1 protein produced a 42.1% ML burden reduction (Bi et al., 2015) and Salmonella-delivered *Ts*87 DNA conferred a 34.2% ML burden reduction against challenge (Yang Y. et al., 2010). Two possible reasons might explain the higher protective efficacy of rMEP observed in the present study. Firstly, balanced cell-mediated and humoral immune responses induced by rMEP may contribute to the protective immunity. Our study showed that immunization with rMEP induced high levels of specific IgG and a mixed IgG1/IgG2a response (**Figure 3**), with an isotype ratio (IgG1/IgG2a) > 1.0, indicative of a Th2-dominated isotype response (Mosmann and Coffman, 1989). In addition, immunization with rMEP induced strong splenocyte proliferation and high levels of Th1 cytokine (IFN-γ) and Th2 cytokines (IL-4, IL-5) upon stimulation of rMEP (**Figure 4**). The stimulation with individual epitope peptide also showed increased T cell proliferation for all four T cell epitopes than the negative control, indicating these four T cell epitopes were presented effectively *in vivo* and stimulated cellular immune responses (**Figure 4**). Cytokines profiles results showed that three of four individual T cell epitopes (P2, P3, and P4) induced elevation of Th2 cytokines (IL-4 and IL-5). The possibility for impotency of P5 in cytokine induction presumably is that P5 was constructed at the end of T epitope array that may be folded inside of the structure and prevented its processing. The second reason for the better protective immunity induced by rMEP than its parental r*Ts*-Pmy is that whole r*Ts*-Pmy antigen may contain epitopes involved in host immune suppression. Trichinella and other nematode infection induce strong CD4^+^CD25^+^ regulatory T cell response characterized by high level of IL-10 and TGF-β (Beiting et al., 2007), possibly through secreting some proteins with immunomodulatory function to suppress host immune response as a survival strategy. Mice immunized with *Ts*-Pmy DNA vaccine induced a strong IL-10 response (Wang et al., 2016). Immunization of r*Ts*-Pmy protein also induced CD4^+^CD25^-^Foxp3^+^ T cell population associated with high levels of IL-10 and TGF-β, possibly through stimulating dendritic cells (Guo et al., 2016). *Ts*-Pmy also directly binds to human complement components C8/C9 (Zhang et al., 2011) and C1q (Sun et al., 2015) to inhibit the activation of human complement. These studies indicate that *Ts*-Pmy itself may play a direct role in the suppression of host immune response to Trichinella infection and to the immunization of vaccine antigen as well. Epitope-based subunit vaccine design should take the T cell subsets primed during immunization into consideration and circumvent potential regulatory T cells activation that can handicap efficacy (Moise et al., 2014). In this study, CD4^+^ T cell epitopes that could induce effective Th1/Th2 immune responses were selected to construct rMEP. *Ts*-Pmy did not induce comparable immunity as rMEP did, one possible reason is that only a few immunodominant epitopes in *Ts*-Pmy are sufficient to induce a protective response and the whole protein may contain some epitopes with adverse effect as a vaccine.

In conclusion, a novel multi-epitope vaccine (MEP) was constructed by using four CD4^+^ T cell epitopes and one B cell epitope from *Ts-*Pmy as an array separated with a bi-lysine spacer between each epitope. The MEP was expressed as a soluble rMEP in *E. coli*. Mice immunized with rMEP vaccine produced significant higher muscle larval reduction (55.4%) than that induced by the immunization of parental r*Ts*-Pmy (34.4%) against *T. spiralis* infection. The higher protection is associated with rMEP induced high levels of anti-rMEP specific IgG and subclass IgG1/IgG2a, elevated splenocytes T cell proliferation and secretion of IFN-γ, IL-4 and IL-5. The cytokine profiles and T cell proliferation of splenocytes from mice immunized with rMEP upon stimulation of individual T cell epitope suggested that most of T cell epitopes are exposed and processed well during immunization that may contribute to the high protection induced by the immunization of rMEP. This study implies that epitope vaccine is a promising approach for the development of vaccines against Trichinellosis. In future studies, a multi-epitope vaccine containing more protective epitopes from other vaccine candidates will be designed in order to induce more comprehensive protective immunity. In addition, as different adjuvants may induce different immune responses (Wilson K.L. et al., 2015), other adjuvants inducing Th1 and/or Th2 responses will be attempted to produce better protective immunity of rMEP.

## Author Contributions

YG designed and performed the experiments; XS, BL, and JH helped with the animal experiments. YG wrote the paper; BZ and XZ gave critical reviews of the paper.

## Conflict of Interest Statement

The authors declare that the research was conducted in the absence of any commercial or financial relationships that could be construed as a potential conflict of interest.
